# Medical students' attitudes toward lumbar puncture—And how to change

**DOI:** 10.1002/brb3.1310

**Published:** 2019-05-06

**Authors:** Moritz von Cranach, Tilo Backhaus, Jochen Brich

**Affiliations:** ^1^ Department of Neurology and Neuroscience Medical Center – University of Freiburg Freiburg Germany

**Keywords:** lumbar puncture, medical students, simulation training

## Abstract

**Objective:**

To survey medical students on the lumbar puncture (LP) procedure in terms of their existing knowledge, practical experience and attitudes, and to determine whether the completion of a single standardized seminar that includes practical training on phantoms can alter these parameters.

**Methods:**

The survey was completed by medical students undertaking the curricular neurology course. Students were asked to describe their practical experience in different bedside procedures, and document how they perceive LP in terms of their own knowledge, confidence and attitude. Students then participated in a newly designed 90‐min seminar that included practical training on phantoms and placed special emphasis both on the patients' point of view during the procedure and the benefits of an atraumatic approach. All students who completed the seminar were required to complete the survey for a second time.

**Results:**

Among the 153 participants, LP was associated with the lowest baseline levels of experience and confidence compared to other bedside procedures. Attitudes, knowledge, and confidence related to the various aspects of LP all showed significant improvement after the seminar.

**Conclusion:**

A single standardized LP seminar with simulation training alters medical students' attitudes toward LP through improving their level of knowledge and confidence. This may have important implications in doctors‐to‐be on their stance toward LP and resultant advice to future patients regarding this important procedure.

## 1 INTRODUCTION

Lumbar puncture (LP) is an essential tool in daily clinical practice, particularly for diagnostic approaches to infectious and demyelinating diseases of the central nervous system in the field of neurology, but also in anesthesiology, oncology, or geriatric medicine (Roos, 2003). Although it is regarded as a relatively safe procedure (Alcolea et al., 2014; Duits et al., 2016), a negative attitude toward LP appears to prevail in the general population (Borhani‐Haghighi, Rezaei, Etemadi, Ghaem, & Shariat, 2009; King & Rwegerera, 2015; Tsvetkova et al., 2017). Explaining this procedure to patients in a way that promotes understanding and acceptance thus requires the health care professional to have a positive attitude toward LP, based on specific knowledge and confidence. Little is known about the attitude of medical students toward LP. Data from the US show that medical students associate LP with the highest levels of perceived difficulty but the lowest levels of self‐confidence compared to equivalent multistep bedside procedures (Dehmer et al., 2013; Wu, Elnicki, & Alper, 2006, 2008). Hence, it is conceivable that such an appraisal—if persistent until residency—can negatively influence patients' attitudes as well as their willingness to undergo LP. These attributes may be reinforced by inherent practical experience, but skill acquisition and practical training in multistep bedside procedures such as LP remain underrepresented in medical education (Barr & Graffeo, 2016). As a consequence, international competency‐based curricula have begun to advocate the introduction of these procedures into medical schools (Bürgi, 2008; Fischer, Bauer, & Mohn, 2015; Merlin, Horak, Milligan, Kraakevik, & Ali, 2014; The Scottish Doctor, 2011), with a general recommendation for initial simulator‐based training (Barsuk et al., 2012; McGaghie, Issenberg, Cohen, Barsuk, & Wayne, 2011).

The objectives for this study were therefore to survey German medical students about the LP procedure in terms of their existing knowledge, practical experience, and attitudes, and determine whether the completion of a single standardized seminar that includes practical training on phantoms can influence these parameters.

## 2 METHODS

### 2.1 General context

This study was conducted in 2016 at the Department of Neurology and Neuroscience, University Medical Center Freiburg. Study participants were 4th, 5th and 6th year medical students (*n* = 153) who partook in the curricular 6‐week clinical neurology course. The course consisted of lectures, team‐based learning units, seminars (including patient presentation) and bedside teaching.

### 2.2 Study design

The majority of participants in this interventional study voluntarily took part in a newly designed and implemented 90‐min LP seminar. Endpoints were attitudes and self‐reported confidence and knowledge levels before and after the seminar. Participation in this study was voluntary and anonymous.

### 2.3 Standard protocol approvals and registrations

This study was approved by the research ethics committee of the University of Freiburg Medical Center (Application number 10001/18).

### 2.4 Questionnaires

**Figure 1:**
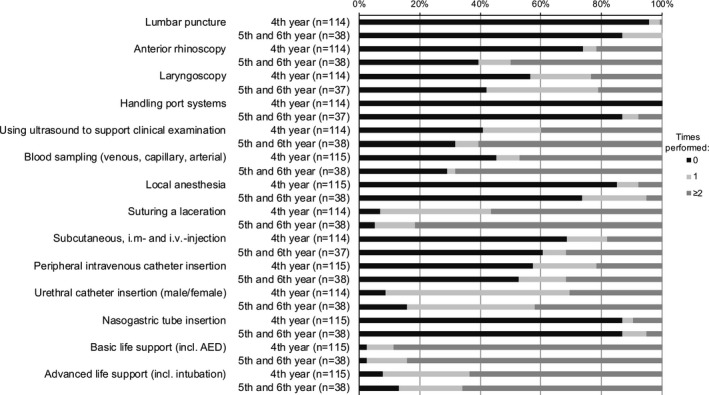
Self‐assessment of experience with different procedures on phantoms. Distribution of the three categories: never performed, performed once, performed two or more times

**Figure 2:**
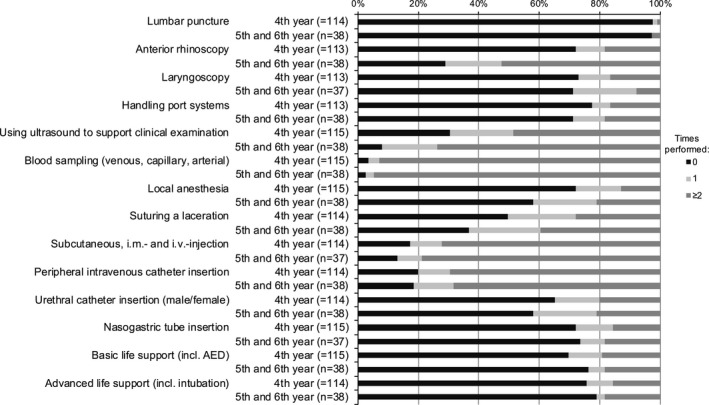
Self‐assessment of experience with different procedures on patients. Distribution of the three categories: never performed, performed once, performed two or more times

**Figure 3:**
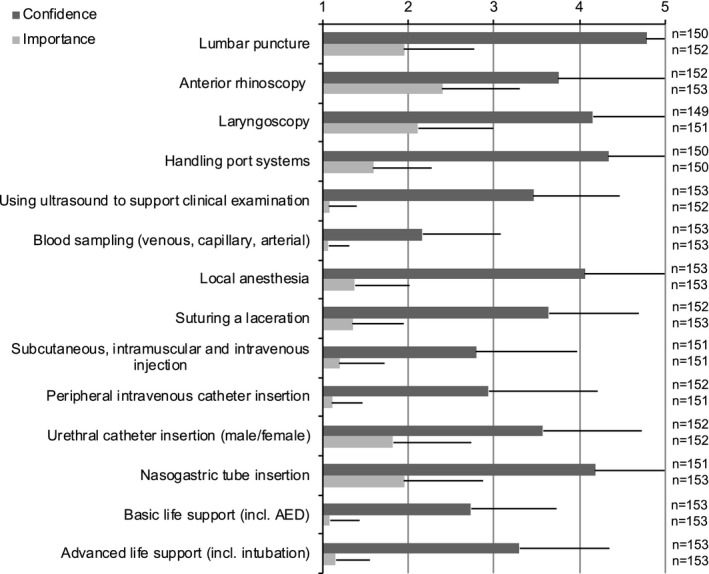
Confidence in and perceived importance of various skills and procedures. 1= very confident/very important, 5 = not confident/not important. Mean + *SD*

Data collection was accomplished via two questionnaires using 5‐point Likert scales. The first questionnaire (prequestionnaire), which was distributed at baseline to all participants of the mandatory curricular course between 1 and 2 weeks before the LP seminar, began with the self‐assessment of competence levels, knowledge, and perceived importance of various clinical skills and procedures (on phantoms and patients); this information was based on a compilation by Wu et al., (2006, 2008) and adapted to Chapter 14b of the German National Competence‐based Learning Objectives Catalogue for Undergraduate Medical Education (Fischer et al., 2015). The resulting lists are presented in Figures 1, 2, 3.

The second questionnaire (postquestionnaire) that was distributed directly after the voluntary LP seminar repeated the aforementioned questions about knowledge, skills and attitudes, and comprised an evaluation based on the "Trier Inventory for Teacher Effectiveness Evaluation" (Krampen & Zayer, 2003, 2006) (Items 1–13), with five additional items focusing on specific aspects of LP. Students were then asked to rate the seminar with a final overall grade.

### 2.5 Small‐group seminar with practical training on phantoms

The 90‐min small‐group seminar (max. six participants) was especially designed for this study and held by neurologists with extensive clinical and teaching experience. The slides were developed based on fundamental didactic principles such as clearly formulated learning objectives, worked examples, visualization of algorithms, and concepts as teaching strategies (Hattie, 2008). In addition, Mayer's principles of multimedia design were applied (Mayer, 2010). The first 45 min consisted of a standardized theoretical overview of various aspects of LP including indications, contraindications, risks, complications and their management, needle types, and analysis of cerebrospinal fluid using projected slides. This part of the seminar was concluded with a step‐by‐step checklist for executing the LP, based on the LP Guideline of the German Neurological Society (Woitalla, 2012). An extra section covered nontechnical aspects such as the creation of an adequate environment and empathetic communication. The ensuing practical part of the seminar was based on Peyton's teaching approach to small groups (Peyton, 1998). After a standardized demonstration by the instructor, each participant had between 15 and 20 min to perform the LP procedure on phantoms (Spinal Simulator 1, 3B Scientific, Hamburg, Germany) placed in sitting and lying positions. The procedure was carried out according to the checklist mentioned above, using original LP equipment including atraumatic 22 gauge spinal needles. It took place under instructor supervision and with peer feedback. Instructors were required throughout the seminar to invite the students to reflect on the patients' situation before, during and after the LP.

### 2.6 Statistical analysis

Statistical analysis was performed using PAST (2016, Oslo, Norway). Likert scale results were treated as cardinally scaled items and evaluated by comparing their means and standard deviations (Carifio & Perla, 2008). To uncover differences, the Mann‐Whitney‐*U* Test on mean Likert Scores was applied with a significance threshold of *p* < 0.05. Missing data were documented as such in the database and in the tables and figures. In case of missing data for particular analyses, the respective subsets were excluded and the specific number of included participants was reported. Because of multiple testing in the same sample, all original *p*‐values were tested for false discovery rate using Bonferroni–Holm and Benjamini–Hochberg procedures with significance levels set at *p* < 0.05. All individual *p*‐values were smaller than their corresponding alpha‐threshold, which corroborates the statistical significance.

## 3 RESULTS

Among the 153 students who completed the prequestionnaire (96% of all students of the course), 115 (75.2%) were in their 4th year, 35 (22.9%) were in their 5th year and three (1.9%) were in their 6th year of medical school (mean 4.27 ± 0.49 years). In the practical skills section of the prequestionnaire, LP was the second most infrequently performed procedure on phantoms, and the least performed on patients, both for 4th year and 5th/6th year students (Figures 1 and 2).

Students felt the least confident in performing LP (4.78 ± 0.55 on a 5‐point Likert Scale: 1 = very confident, 5 = not confident) in comparison to the other skills and procedures listed, although LP was perceived as important (1.95 ± 0.82 on a 5‐point Likert Scale: 1 = very important, 5 = not important, Figure 3).

**Figure 4:**
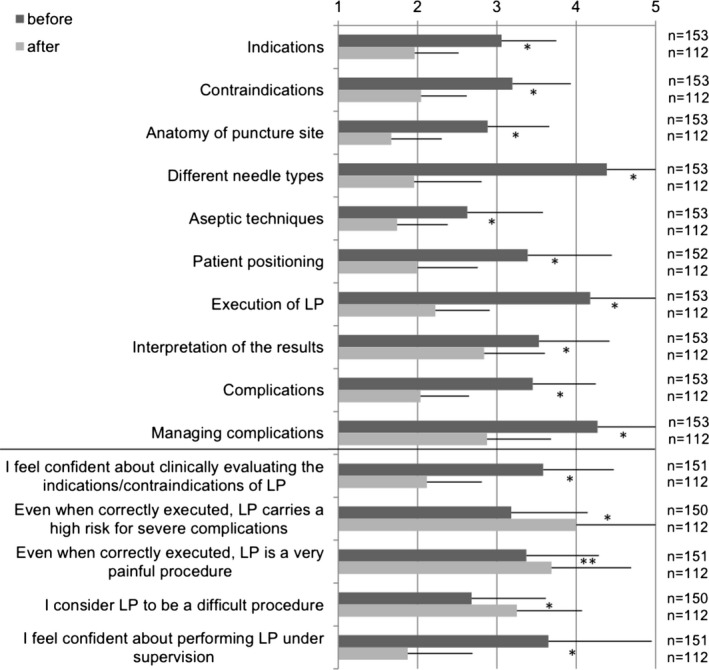
Students' attitudes, knowledge, and confidence regarding lumbar puncture before and after the seminar. 1 = very confident/strongly agree, 5 = not confident/strongly disagree. Mean + *SD*. *p < 0.001, **p = 0.03

Students were next required to carry out a self‐assessment of their knowledge and skills in relation to different aspects of LP, with five additional questions addressing their attitude toward LP (Figure 4).

The postquestionnaire containing follow‐up questions was obtained from 112 students (73.2%), the postseminar evaluation from 109 students (71.2%). Figure 4 shows the degree of perceived knowledge and skills, as well as confidence levels and attitudes in relation to LP before and after the tutorial seminar. After the seminar, knowledge and confidence levels as well as attitudes toward risk, pain, and difficulty improved significantly in every category compared to that before the seminar (Item “Pain level” *p* = 0.03, all other *p* < 0.001).

The results of the student evaluation revealed very high levels of appreciation and satisfaction, not least regarding a better understanding of the patients' situation during LP. The overall grade was 1.13 (1 [highest] to 6 [lowest], Table 1).

**Table 1:** Student evaluation of the seminar

		Mean	*SD*
1	The learning objectives were clear and comprehensible	1.08	0.31
2	The learning content of the teaching unit was adjusted to the learning targets	1.17	0.40
3	The targeted learning objectives were achieved	1.27	0.50
4	The instructor was always well prepared	1.17	0.52
5	Didactic tools (i.e. slides) were used in an adequate way	1.21	0.56
6	The instructor was able to explain difficult learning content in an understandable way	1.21	0.49
7	The instructor appeared committed to the seminar	1.14	0.37
8	The style of speech used by the instructor was fluent and clear	1.12	0.45
9	I was motivated to follow the topic during the seminar	1.20	0.51
10	The seminar had an atmosphere conducive to student contributions	1.06	0.28
11	An adequate number of discussions took place	1.36	0.60
12	Student discussions were efficient	1.44	0.64
13	Questions and contributions were always welcome by the instructor	1.06	0.28
14	The seminar helped me to better understand the indications and contraindications of LP	1.19	0.42
15	The seminar helped me to better understand the risks and complications of LP	1.28	0.51
16	The practical simulation on phantoms was very helpful	1.25	0.53
17	The seminar contributed to a better understanding of the patient's situation during LP	1.39	0.59
18	Overall, the seminar improved my understanding of LP	1.14	0.37
19	Please assign this seminar an overall grade	1.13	0.34

Note

*N* = 109; Items 1–18 are mean values representing the students' evaluation scores: 1 = "I completely agree”, 5 = "I completely disagree.” Item 19 represents the overall grade: 1 = highest (“excellent”) to 6 = lowest (“failure”).

Abbreviation: LP, lumbar puncture.

## 4 DISCUSSION

Lumbar puncture was ranked by our medical students as the procedure in which they had the least confidence and experience, both in simulated and real‐life settings, even though it was rated as important. These results are in line with those of similar studies in the US (Dehmer et al., 2013; Wu et al., 2006, 2008). For the first time, we also present data about the attitudes of medical students toward LP. Before the seminar, students classified LP as a difficult and critical procedure, which, even when correctly executed, is not only rated painful but also associated with a high risk for severe complications. This is in strong contrast to a recently established expert opinion, which stated that “When an LP is performed correctly, the procedure is well tolerated and accepted with a low complication rate” (Engelborghs et al., 2017). Although we did not directly ask about the reasons for the negative appraisal of our students, recent research by Henriksen et al. (2017) provides insight into some of the thoughts experienced by physicians before they perform a LP, for example, fear of injuring the medulla due to puncturing the wrong area, or anxiety about the proximity of the meninges before and during the LP. To make matters worse, they also seem to worry about hurting the patient because of the “blind” deep insertion of a long needle into the back.

These findings of negative attitudes indicate that there is a need for more information and understanding of the procedure LP in medical students. Simulation‐based LP training might be a self‐evident way for this. We therefore designed a seminar with practical training on phantoms to address these concerns: In the theoretical part of our seminar we extensively discussed in a small‐group setting both the indications and the contraindications for LP, while incorporating the neuroanatomical and physiological background of the technique. In their role as instructors, this allowed the experienced neurologists to clear up any misconceptions, while simultaneously providing the students with explicit guidelines for properly executing a LP. The presentation of possible complications and their prevention and management further helped students to reduce their negative attitudes toward the LP procedure. In addition, revising the neuroanatomy of the lumbar region as well as discussing the need for optimal positioning helped the students to appreciate the possible sources of local pain and, under the guidance of the experienced instructor, to learn how to best handle these situations. The results of the seminar evaluation and the postquestionnaire demonstrate that these learning goals were accomplished in a highly positive way and helped positively influencing the students' attitudes toward the LP.

Another important element of uncertainty for the medical students was the distinguished use of different needle types in LP: Our students initially disclosed a great lack of confidence in this important decision to be made for every LP. We therefore included a discussion about different needle types and their consequences for the likelihood of one of the most common complications of LP, the postdural‐puncture headache in our seminar. Given the persistent widespread use of traumatic needles (Duits et al., 2016; Moisset et al., 2016)—most likely the result of being passed down through a traditional top‐down teaching system (Tung, 2013)—our seminar enabled us to demonstrate the advantages of atraumatic needles for diagnostic LP using convincing scientific data (Nath et al., 2018) and to teach the handling of the atraumatic approach from the very beginning. These interventions led to a remarkable increase in the students' knowledge about needle types, thereby setting the cornerstone for a primary atraumatic approach to LP. This is especially promising, given the recent demonstration of a long‐term effect of a teaching intervention used in residents to foster the atraumatic approach (Tung, 2013).

The practical part of our seminar was intentionally kept simple, without the inclusion of a context‐rich case to reduce cognitive load upon the first encounter with LP (Young, Merrienboer, Durning, Cate, & Cognitive, 2014); this allowed the focus to stay on the multistep LP procedure itself. The didactic approach to the practical part of the seminar was adapted from the well‐established Peyton approach (Peyton, 1998). This not only ensured that the instructor performed a “gold standard” LP demonstration, but also enabled constructive and useful peer feedback for the students' own attempts (Shanks, Brydges, Brok, Nair, & Hatala, 2013). Students deemed this type of practical simulation as very helpful. Taken together, all of these points accounted for the significantly positive change in students' self‐assessed levels of confidence in performing a LP under supervision, although from our perspective this was only a secondary learning objective of the seminar. In this regard, it is noteworthy that we did not check for success of the LP training using a checklist or other types of tests, since we are well aware of the fact that a multistep procedure like the LP cannot be mastered after training for <1 hr. Mastery learning approaches in later phases of education matched to the disciplines of LP application might be an appropriate way for this task (Barsuk et al., 2012).

Another important learning objective of the seminar was to promote an understanding of the patient's situation during an LP. Based on the facts that the procedure is performed behind the patient's back and patients are known to experience anxiety (Borhani‐Haghighi et al., 2009; Duits et al., 2016; King & Rwegerera, 2015) about having a LP, the recent “Consensus guidelines for LP in patients with neurological diseases” (Engelborghs et al., 2017) stated that: “As fear of the LP and post‐LP complications can be influenced by the attitude of the physician and nursing staff and can be decreased by giving reassuring but adequate information, it is of most importance to carefully inform the patient.” Moreover, “During the LP procedure, it is important to explain the different steps of the LP procedure, thereby reducing eventual anxiety and discomfort […] (Level II‐2 evidence).” Throughout the seminar, students were encouraged to reflect upon the patients' situation before, during and after the LP, with helpful tips from the instructor contributing to a better understanding of this aspect.

There are several limitations to this study. Firstly, it was not tested whether the positive, self‐assessed changes to the levels of attitude, knowledge and confidence in performing an LP under supervision actually translated to better performance in real life. Further studies are needed to investigate these effects in real life. Although we could not test for the long‐term retention of our results, related studies have demonstrated a perpetuation of initially set standards for bedside procedures, both in general (Garrood et al., 2010), and for LP (Tung, 2013).

About 27% of students attending the curricular neurology course did not participate in our voluntary seminar, reducing the number of postquestionnaires that could be analyzed and hence introducing a possible selection bias. The reasons for nonattendance are not known. Nevertheless, we believe that the absolute number of 112 postquestionnaires is sufficient for analysis since we had a quite homologous group, which is reflected by the analysis of the results of the final summative examination of the course: 134 of the 153 participants (more than 87%) achieved the grades “very good” or “good”, with 14 achieving “satisfactory” and only four achieving “sufficient” (0 failed) (data not shown). Since pre‐ and postquestionnaires were not paired, comparison was accomplished between means rather than individual values and without a pairing process. This limitation is also attenuated by the homogeneity of the group.

Finally, as a single center study without a control group, institutional confounders could not be ruled out, although baseline results did not differ greatly from comparable studies.

In summary, we have demonstrated that a single standardized LP seminar with simulation training can positively change the initially negative attitudes of medical students toward LP by improving their knowledge of and confidence in this important procedure.

Since this type of seminar is still not routinely implemented in German curricula (Isenmann, Biesalski, Zupanic, & Gerloff, 2013), we strongly advocate for the implementation of such LP seminars: The changes of attitudes evoked in these seminars may have important implications in doctors‐to‐be on their stance on LP and resultant advice to future patients and thereby could contribute to an improvement in the reputation of the LP procedure.

## CONFLICT OF INTEREST

None of the authors declare conflict of interests.

### DATA AVAILABILITY STATEMENT

The data that support the findings of this study are available from the corresponding author upon reasonable request.
